# A novel *CD40LG* frameshift mutation in an Iranian family with X-linked hyper-IgM syndrome: A case report

**DOI:** 10.1016/j.ymgmr.2026.101376

**Published:** 2026-10-17

**Authors:** Fatemeh Farzi, Mohammad Panahi, Ebrahim Sakhinia, Effat Alizadeh

**Affiliations:** a Department of Medical Biotechnology, Faculty of Advanced Medical Sciences, Tabriz University of Medical Sciences, Tabriz, Iran; b Laboratory for Molecular Genetic Diagnostics, Institute of Human Genetics, University of Heidelberg, Heidelberg, Germany; c Department of Medical Genetics, Faculty of Medicine, Tabriz University of Medical Sciences, Tabriz, Iran; d Department of Laboratory and Regenerative Medicine, University of Manchester, Manchester, United Kingdom

**Keywords:** Hyper-IgM syndrome type 1, CD40LG, Novel frameshift mutation, Whole-exome sequencing, Prenatal diagnosis

## Abstract

**Background:**

Pathogenic mutations in the CD40LG gene lead to X-linked hyper-IgM syndrome type 1 (X-HIGM1), a rare primary immunodeficiency marked by impaired class-switch recombination of immunoglobulins and frequent severe infections. Early molecular diagnosis is essential for appropriate management, genetic counseling, and prenatal diagnosis.

**Case presentation:**

We investigated a 5-year-old Iranian boy with recurrent otitis media and pneumonia since infancy, progressive inflammatory arthritis, and a striking family history of early male deaths suggestive of an inherited X-linked immunodeficiency. Baseline immunological evaluation before initiation of IVIG showed markedly reduced IgG (2 mg/dL) and undetectable IgA (0 mg/dL), while IgM remained within the reference range (114 mg/dL). Lymphocyte subset analysis showed no major abnormality in the distribution of conventional T-cell subsets. Whole-exome sequencing (WES) revealed a hemizygous frameshift mutation in CD40LG, c.127del (p.Ala43LeufsTer11), predicted to introduce a premature termination codon and result in loss of normal CD40LG function. Sanger sequencing confirmed the variant in the proband and identified his mother as a heterozygous carrier, with the segregation pattern in the tested family members consistent with X-linked inheritance. Prenatal testing further showed that the fetus did not carry the pathogenic variant.

**Results:**

According to ACMG/AMP criteria, the c.127del variant was classified as pathogenic based on predicted loss of function, absence from population databases, phenotype specificity, and segregation evidence (PVS1, PM2_supporting, PP1_supporting, PP4). This variant affects exon 1 of CD40LG, a less commonly reported region compared with the known mutational hotspot in exon 5.

**Conclusion:**

We report a novel pathogenic *CD40LG* frameshift variant associated with X-HIGM1 in an Iranian family. This report broadens the CD40LG mutation spectrum and underscores the utility of WES for confirming diagnosis, identifying carriers, enabling genetic counseling, and directing prenatal diagnosis for families with suspected X-linked immunodeficiency.

## 1 Introduction

Immunoglobulin isotype switching is a critical process in adaptive immunity that requires coordinated signaling through cytokines and direct T–B cell interactions [1]. Primary immunodeficiency disorders (PIDs), including hyper-IgM (HIGM) syndromes, are rare but potentially life-threatening conditions characterized by defects in this process [2]. Alterations in the CD40LG gene (also known as CD40L, TNFSF5, or gp39) lead to X-linked hyper-IgM syndrome (X-HIGM1). This gene produces an essential ligand expressed on activated CD4+ T cells that binds to the CD40 receptor present on B cells, macrophages, and dendritic cells [1], [3]. Interruption of this signaling pathway disrupts class-switch recombination, leading to normal or increased serum IgM levels alongside decreased IgG and IgA concentrations [2], [4]. Consequently, affected individuals tend to experience repeated episodes of infections including otitis media, sinusitis, bacterial pneumonia, and Pneumocystis jirovecii pneumonia [3], [5]. In addition, patients may develop autoimmune diseases, neutropenia, liver disease (sclerosing cholangitis), and malignancies such as lymphoma [1], [5], [6].

The human CD40LG gene is located on the X chromosome at Xq26.3 and comprises five exons and four introns [7]. It encodes a 261-amino-acid type II transmembrane protein consisting of an N-terminal intracellular tail (residues 1–22), a transmembrane segment (residues 23–46), an extracellular region (residues 47–122), and a C-terminal TNF-homology (TNFH) domain (residues 123–261) [7]. Exon 1 encodes the intracellular and transmembrane regions and a small portion of the extracellular region, whereas exons 2–5 encode the remainder of the extracellular region. The TNFH domain is encoded predominantly by exon 5 and partly by exon 4, and exon 5 contains a high proportion of reported pathogenic variants [6], [8]. Diagnosis of X-HIGM1 is supported by characteristic clinical and immunological findings and can be further confirmed by molecular genetic testing. CD40L expression on activated CD4+ T cells can also be evaluated by flow cytometry as a functional diagnostic test; however, this assay was not available for the present patient. In such circumstances, the combination of the clinical phenotype, immunological findings, and identification of a pathogenic CD40LG variant can provide strong evidence for the diagnosis [2], [3]. Hematopoietic stem cell transplantation (HSCT) is a potentially curative treatment option for CD40L deficiency [9].

In this study, we investigated the molecular basis of disease in a patient with clinical features suggestive of X-HIGM1 and identified a novel *CD40LG* mutation, thereby expanding the mutational spectrum of *CD40LG*.

## 2 Materials and methods

### 2.1 Genetic counseling and diagnostic approach for hyper-IgM syndrome

Patients with suspected primary immunodeficiency were referred to the medical genetics laboratory for further evaluation. One family was identified in which the underlying genetic cause of the suspected immunodeficiency had not been established using conventional diagnostic approaches. The proband had initially been diagnosed with juvenile idiopathic arthritis (JIA) because of his inflammatory joint manifestations. Subsequently, recurrent infections and abnormal immunological findings raised suspicion of an underlying primary immunodeficiency (PID), prompting further genetic evaluation. The family was subsequently referred to a genetic diagnosis center for further molecular investigation and genetic counseling regarding the risk of recurrence in future pregnancies. A comprehensive medical history was obtained, and the relevant pedigree was constructed (Fig. 1). Whole-exome sequencing (WES) was recommended to identify the underlying molecular cause of the suspected inherited immunodeficiency. The proband was identified as individual IV-6 in the pedigree. Blood samples were obtained from the proband, his mother, and an unaffected brother for genetic analysis and segregation assessment.

**Fig. 1:**
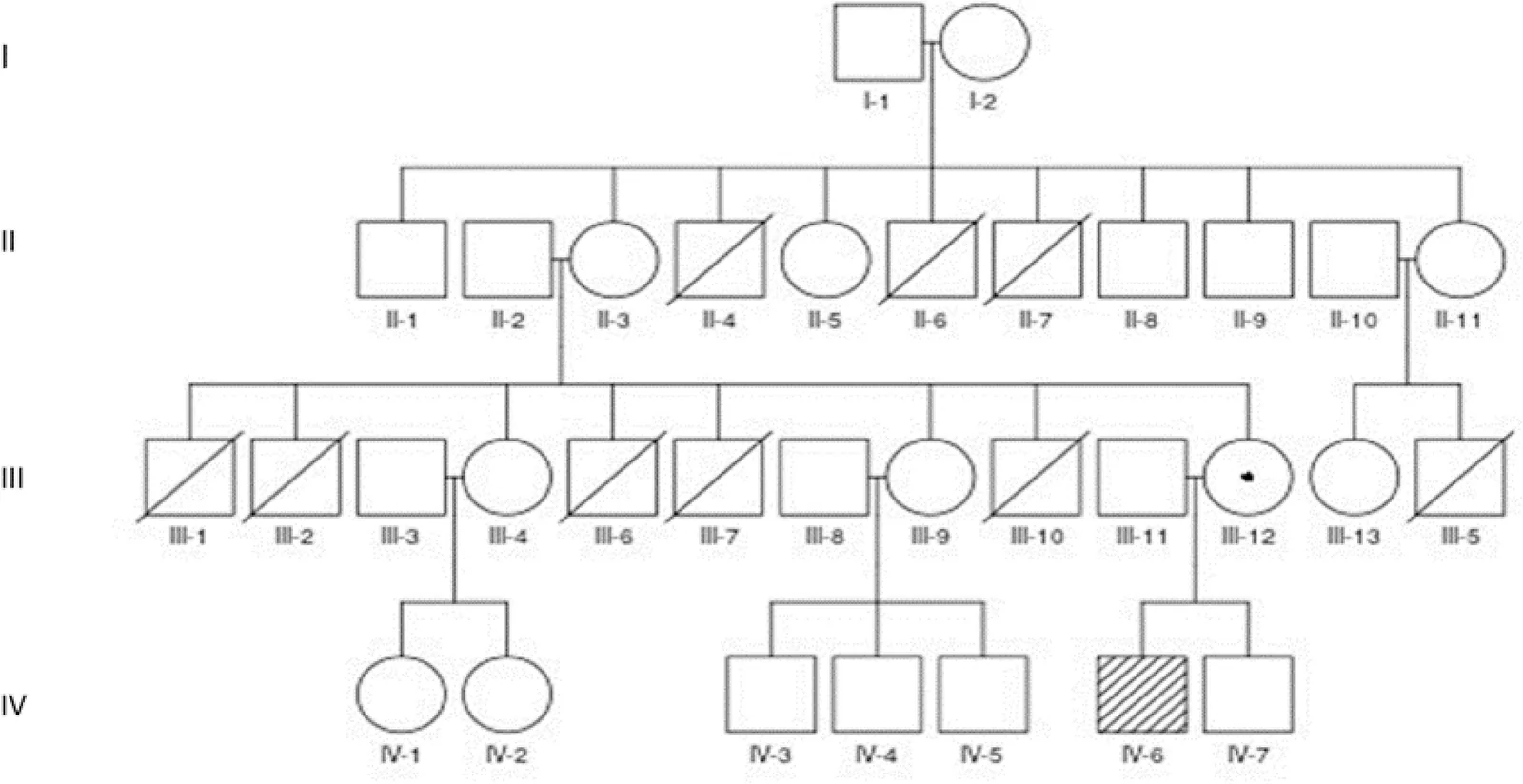
Pedigree of a family with a history of immunodeficiency. Filled symbols indicate affected individuals. White symbols indicate family members without reported immunodeficiency-related symptoms. A dot within a symbol indicates a molecularly confirmed carrier. Carrier status was assigned only to individuals with molecular confirmation; individuals who had not undergone genetic testing were not classified as carriers based solely on family history.

### 2.2 Ethical statement and sample collection

Written informed consent for genetic testing and publication of the patient's clinical and genetic information was obtained from the patient's parents/legal guardian. Genomic analysis was performed on peripheral blood samples collected from the proband and participating relatives in EDTA tubes. During the course of the investigation, the proband's mother (III-12) became pregnant; consequently, amniocentesis was performed by an experienced obstetrician-gynecologist to obtain an amniotic fluid sample for prenatal genetic diagnosis.

### 2.3 Whole-exome sequencing

Whole-exome sequencing (WES) was performed on genomic DNA extracted from peripheral blood samples. The extracted DNA was fragmented, followed by adapter ligation and barcoding, and subsequently subjected to solution-phase hybridization for target enrichment using the Agilent SureSelect Human All Exon V7 Plus probe set. Next-generation sequencing was performed on the Illumina NovaSeq 6000 platform using paired-end sequencing. The exomes were sequenced to an average target coverage of approximately 100×, with 90–95% of the targeted bases achieving a sequencing depth of at least 20×. The paired-end reads were aligned to the NCBI human reference sequence (GRCh37) using the Burrows-Wheeler Aligner (BWA), and variant calling was performed using the Genome Analysis Toolkit (GATK). Variants were subsequently filtered based on their population frequency, predicted functional consequences, and known disease associations. Variants with a minor allele frequency (MAF) ≤1% in the NHLBI Exome Sequencing Project (ESP), variants classified as disease-causing in established public databases, and predicted loss-of-function variants with a MAF ≤1% were prioritized for further evaluation. The potential contribution of each prioritized variant to the patient's phenotype was assessed by integrating genetic, clinical, and immunological findings, and candidate variants were classified according to the American College of Medical Genetics and Genomics (ACMG) criteria. Only variants with sufficient evidence supporting a potential role in the patient's disease phenotype were considered for clinical interpretation and reporting.

### 2.4 Variant classification

Variants were classified according to the ACMG/AMP guidelines [10], and evidence strengths were additionally evaluated using the ACMG/AMP point-based framework [11].

### 2.5 Whole-exome sequencing data analysis

The raw FASTQ files were processed using the Genomize bioinformatics platform (version 16.1). Initial quality assessment was performed on the raw reads, followed by alignment to the human reference genome (GRCh37/hg19) using the Burrows-Wheeler Aligner (BWA). The alignment output was generated in Sequence Alignment Map (SAM) format and subsequently converted to Binary Alignment Map (BAM) format. PCR duplicates were removed, and base quality score recalibration (BQSR) was applied to the data.

Variant discovery, including single-nucleotide variants (SNVs) and small insertions/deletions (indels), was performed utilizing the Genome Analysis Toolkit (GATK) HaplotypeCaller. For the functional annotation of identified variants, the ANNOVAR tool was employed, integrating data from several public databases, including dbSNP, the Exome Variant Server (EVS), the 1000 Genomes Project (1KGP), and the Exome Aggregation Consortium (ExAC).

A minor allele frequency (MAF) cutoff of less than 1% in the general population was used to filter variants, thereby prioritizing rare alleles. Synonymous variants were excluded, whereas variants affecting consensus splice sites or resulting in non-synonymous amino acid changes were retained. To evaluate the pathogenic potential of the candidates, additional resources, including avsnp150, dbNSFPv42c, dbscSNVv11, and the UCSC Genome Browser, were utilized. Variant filtering and interpretation were performed using established approaches previously applied in clinical exome sequencing studies [12], [13], followed by variant classification according to the ACMG/AMP guidelines [10]. To assess whether the identified CD40LG variant had been previously reported, additional searches were performed in ClinVar, gnomAD, Iranome, LOVD, HGMD, and the Exome Variant Server (EVS), as well as in the published literature indexed in PubMed. The variant was queried using the HGVS descriptions c.127del and p.Ala43LeufsTer11; genomic coordinates were also used for the Iranome search. These searches were conducted in October 2026. To the best of our knowledge, no previous record of the c.127del (p.Ala43LeufsTer11) variant was identified in the queried databases or published literature.

### 2.6 Validation of sanger sequencing and analysis of co-segregation

After the candidate variant was identified through WES, Sanger sequencing was carried out to confirm the mutation and assess its co-segregation pattern across the family. Genomic DNA from the proband, his mother, and his unaffected brother was used for targeted amplification of the affected region in the CD40LG gene. Polymerase chain reaction (PCR) was performed using the following specific primers: F: 5′-CCACTTTGACAGTCTTCTC-3′ and R: 5′-GGTTAGGATCTTGGGATC-3′.

A SimpliAmp Thermal Cycler (Thermo Fisher Scientific, USA) was used for amplification. The resulting PCR products were subsequently purified and sequenced using an ABI 3500XL Genetic Analyzer (Applied Biosystems, USA). The sequencing chromatograms were analyzed and compared with the reference sequence using ChromasPro v2.1.3 software. This analysis was also applied to the fetal DNA obtained via amniocentesis to determine the presence of the familial mutation.

## 3 Results

### 3.1 Clinical presentation and laboratory findings

The proband was a 5-year-old boy who presented with recurrent otitis media and pneumonia beginning in infancy. He subsequently developed progressive inflammatory arthritis involving the finger and knee joints, resulting in significant functional limitation. Because of his inflammatory joint manifestations, he was initially diagnosed with juvenile idiopathic arthritis (JIA). At two years of age, recurrent infections and abnormal immunological findings prompted evaluation for an underlying primary immunodeficiency (PID).

Before initiation of intravenous immunoglobulin (IVIG) replacement therapy, baseline immunological evaluation showed markedly reduced IgG and undetectable IgA, whereas IgM remained within the laboratory reference range; serum IgE was also measured. Approximately 1.5 years after initiation of IVIG replacement therapy, repeat immunoglobulin testing showed an increase in IgG, while IgA remained markedly reduced and IgM remained within the reference range. The corresponding immunoglobulin values before and after IVIG are presented in Table 1. The follow-up IgG measurement was obtained during ongoing IVIG replacement therapy and therefore does not necessarily reflect endogenous immunoglobulin production.

**Table 1:** Immunological and hematological findings before and approximately 1.5 years after IVIG replacement therapy.

Parameter	Before IVIG (2 years)	∼1.5 years after IVIG	Reference range
IgG (mg/dL)	2	461	386–1470
IgA (mg/dL)	0	0.3	25–154
IgM (mg/dL)	114	64	37–224
IgE (IU/mL)	3	2	<280
Neutrophils (%)	22.6	29.1	25–55⁎ / 35–55⁎⁎
Absolute neutrophil count (×10^3^/μL)	0.98	2.71	1.8–7.7⁎ / 1.9–8.0⁎⁎
CD3+ (%)	78	60	28–77
CD4+ (%)	52	36	32–62
CD8+ (%)	21	15	2–36
CD16+ (%)	2.84	–	5–19
CD19+ (%)	15.38	–	3–14
CD20+ (%)	15	–	3–15
CD56+ (%)	2.63	–	2–19

Footnote: The follow-up IgG measurement was obtained during ongoing IVIG replacement therapy and therefore does not necessarily reflect endogenous IgG production.

⁎ Reference range before IVIG.

⁎⁎ Reference range approximately 1.5 years after IVIG.

Before initiation of IVIG, the neutrophil percentage and absolute neutrophil count were below the laboratory reference ranges. Approximately 1.5 years after initiation of IVIG, the neutrophil percentage remained below the corresponding reference range, whereas the absolute neutrophil count was within the reference range (Table 1).

Before initiation of IVIG, lymphocyte subset analysis showed mildly increased CD3+ and CD19+ proportions, decreased CD16+ cells, CD20+ cells at the upper limit of the reference range, and CD56+ cells within the reference range, while CD4+ and CD8+ T-cell proportions were within the reference ranges. A subsequent lymphocyte subset analysis after initiation of IVIG showed CD3+, CD4+, and CD8+ cells of 60%, 36%, and 15%, respectively (Table 1).

The family history was notable for multigenerational early mortality among maternal relatives. Five maternal uncles died at an early age, including three at 6–7 months, one at 4 years, and one at 21 years of age. In addition, six brothers of the maternal grandmother reportedly died during childhood, including three at approximately 3 months and two at 7 years of age. Specifically, individuals III-1, III-2, III-6, III-7, and III-10 died with diarrhea and vomiting, whereas individuals II-4, II-6, and II-7 died with cold-like symptoms, diarrhea, vomiting, and diphtheria. None of these deceased relatives had molecular confirmation of CD40LG deficiency; therefore, their clinical histories were considered suggestive rather than confirmed cases of X-linked hyper-IgM syndrome.

At five years of age, whole-exome sequencing identified a hemizygous c.127del variant in the CD40LG gene, predicted to result in p.Ala43LeufsTer11. The molecular findings established the diagnosis of X-linked hyper-IgM syndrome type 1 (X-HIGM1)**.**

### 3.2 Clinical management and HSCT status

Following the diagnosis of primary immunodeficiency at two years of age, the patient has been receiving monthly intravenous immunoglobulin (IVIG) replacement therapy and nightly co-trimoxazole (5 mL) for infection prophylaxis. His inflammatory arthritis has been managed with naproxen, low-dose prednisolone (2.5 mg daily), and methotrexate (7.5 mg once weekly), together with daily folic acid supplementation. Additional supportive treatments include Asmavit, Osteocare, fluticasone, and montelukast. The patient remains under regular clinical follow-up for monitoring of infectious complications, inflammatory manifestations, and treatment response.

HLA typing was performed as part of the evaluation for hematopoietic stem cell transplantation (HSCT). HSCT was recommended by the treating physicians as a potentially curative treatment; however, a suitable donor has not yet been identified. The patient remains under clinical follow-up while donor identification and transplantation planning are ongoing.

### 3.3 WES analysis of the proband and variant classification

Following variant filtering and prioritization, we identified a hemizygous frameshift variant in the CD40LG gene, c.127del (p.Ala43LeufsTer11), in transcript NM_000074.3 (Table 2). This single-nucleotide deletion alters the reading frame and introduces a premature termination codon in exon 1, predicted to cause nonsense-mediated decay and loss of CD40LG function, consistent with the established loss-of-function mechanism of CD40LG; PVS1 was applied.The c.127del (p.Ala43LeufsTer11) variant has not been documented in individuals with CD40LG-related disorders and is not present in population databases (gnomAD v2.1.1 and v4.1; zero allele frequency), meeting the rare in controls criterion (PM2, supporting evidence). The patient's phenotype was consistent with CD40LG-related X-linked hyper-IgM syndrome. Importantly, immunoglobulin measurements obtained before initiation of IVIG demonstrated markedly reduced IgG (2 mg/dL; reference range, 386–1470 mg/dL), undetectable IgA (0 mg/dL; reference range, 25–154 mg/dL), and IgM within the reference range (114 mg/dL; reference range, 37–224 mg/dL). Together with recurrent respiratory infections and neutropenia, these findings supported the application of PP4. Segregation analysis confirmed the variant in the hemizygous proband and his heterozygous mother, whereas the variant was absent in his unaffected brother. Prenatal testing also demonstrated the wild-type allele in the fetus. Because additional affected or at-risk relatives were unavailable for molecular testing, the segregation evidence was considered limited, and PP1 was therefore applied at supporting strength (PP1_supporting), rather than moderate strength (Fig. 2A).

**Table 2:** The results of WES analysis.

Mutant variant	Effect on protein	Type	Zygosity	ClinVar	Frequency (gnomAD)	ACMG
c.127del	p.Ala43LeufsTer11	Frameshift	Hemizygous	NA	NA	Pathogenic (PVS1, PM2_supporting, PP1_supporting, PP4)

**Fig. 2:**
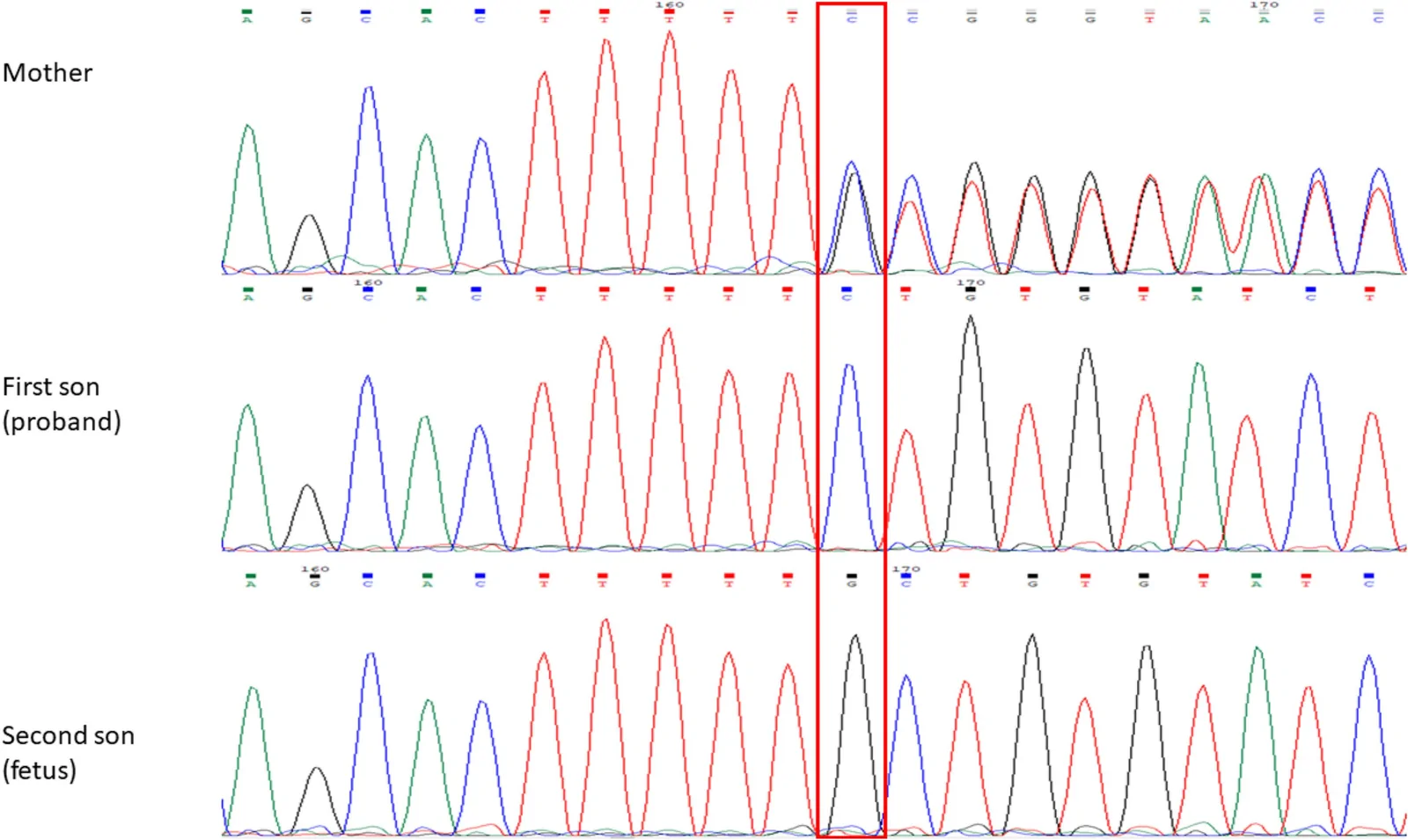
The results of Sanger sequencing for pathogenic variant c.127del in the family.

Following variant filtering and prioritization, we identified a hemizygous frameshift variant in the CD40LG gene, c.127del (p.Ala43LeufsTer11), in transcript NM_000074.3 (Table 2). This single-nucleotide deletion alters the reading frame and introduces a premature termination codon in exon 1, predicted to cause nonsense-mediated decay and loss of CD40LG function, consistent with the established loss-of-function mechanism of CD40LG; PVS1 was applied.

Due to the unavailability of blood samples or genomic DNA from several female family members (I-2, II-3, II-11, III-4, and III-9), variant-site validation by Sanger sequencing could not be performed in these individuals. Therefore, the carrier status of these untested female relatives, including II-3, could not be molecularly confirmed.

In summary, based on ACMG/AMP guidelines and the combined evidence (PVS1, PM2_supporting, PP1_supporting, PP4), the c.127del variant is classified as pathogenic for CD40LG-related HIGM.

### 3.4 Confirmation of the c.127del mutation by sanger sequencing

Sanger sequencing was performed in the available family members to validate the candidate variant identified by WES and assess its segregation in the tested individuals. The analysis confirmed that the patient's mother (III-12) was heterozygous for the c.127del variant, whereas the proband was hemizygous for the variant. The variant was absent in the tested unaffected brother, and these findings were consistent with an X-linked inheritance pattern (Fig. 2). Furthermore, prenatal genetic testing was performed using fetal DNA obtained by amniocentesis. Sanger sequencing demonstrated that the fetus carried the wild-type allele and had not inherited the familial c.127del variant, providing reassurance to the family during the pregnancy.

## 4 Discussion

Hyper-IgM syndrome is a primary immunodeficiency characterized by susceptibility to recurrent infections, particularly respiratory tract infections, with impaired immunoglobulin class switching and reduced memory B-cell development [1], [2]. In this study, whole-exome sequencing (WES) identified a single-nucleotide deletion at cDNA position c.127 in the CD40LG gene, predicted to cause a frameshift and premature termination of the encoded protein. The immunological findings in our patient were consistent with the characteristic pattern of CD40L deficiency, with profound IgG deficiency and undetectable IgA accompanied by preserved IgM before initiation of IVIG replacement therapy. This pattern is consistent with impaired immunoglobulin class-switch recombination, in which IgG and IgA production is markedly impaired while IgM may remain within the normal or elevated range [1], [2]. The follow-up IgG measurement was obtained during ongoing IVIG replacement therapy and therefore does not necessarily reflect endogenous immunoglobulin production.CD40L expression by flow cytometry was not assessed because the assay was not available at the time of evaluation. Therefore, the functional effect of the variant is predicted based on its molecular consequence and the established loss-of-function mechanism of CD40LG, rather than confirmed by direct protein-expression or functional studies.The CD40LG gene encodes CD154, also known as CD40 ligand (CD40L), a type II transmembrane protein expressed primarily on activated CD4+ T lymphocytes [1], [3]. The interaction between CD40L and CD40 is essential for T-cell-dependent humoral immune responses, including immunoglobulin class-switch recombination, germinal center formation, and memory B-cell development [2], [4]. Therefore, impaired CD40L function disrupts CD40-mediated signaling and provides the molecular basis for the impaired antibody responses characteristic of X-linked hyper-IgM syndrome (X-HIGM). X-HIGM typically presents in early childhood with recurrent sinopulmonary infections, Pneumocystis jirovecii pneumonia (PCP), persistent diarrhea, and neutropenia [1], [3], [5]. Autoimmune manifestations, including arthritis, have also been reported in patients with X-HIGM and may reflect underlying immune dysregulation [1], [6]. Our patient presented with features characteristic of X-HIGM, including recurrent respiratory infections and a family history consistent with X-linked inheritance. However, no episode of PCP was documented, whereas inflammatory arthritis was a prominent and disabling clinical manifestation in this patient.

Similar to our case, Li et al. [14]. reported a patient with an exon 1 CD40LG frameshift variant, c.86_87del (p.T29Sfs*18), who presented with severe infectious complications, including Pneumocystis jirovecii pneumonia, cytomegalovirus infection, and fungal infection. In contrast, our patient had recurrent respiratory infections and prominent inflammatory arthritis but no documented opportunistic infection. The c.127del (p.Ala43LeufsTer11) variant identified in our patient represents another frameshift variant affecting exon 1 of CD40LG and further expands the spectrum of reported variants in this region. Unlike the de novo variant reported by Li et al., the segregation findings in our family were consistent with an inherited X-linked pattern. However, these differences should not be interpreted as evidence of a specific genotype–phenotype association, given the limited number of reported exon 1 variants and the clinical heterogeneity of CD40LG deficiency [14].

Several CD40LG variants have previously been reported in Iranian patients with X-linked hyper-IgM syndrome, demonstrating molecular heterogeneity within the Iranian population. Aghamohammadi et al. reported CD40LG and AICDA variants among 23 Iranian patients with HIGM, while Alizadeh et al. identified multiple distinct CD40LG variants in an Iranian cohort [15], [16]. In addition, Torabizadeh et al. reported an exon 5 CD40LG missense variant, c.464 T > C (p.L155P), in an Iranian boy with X-linked hyper-IgM syndrome [17]. However, available Iranian studies comprise relatively small cohorts and individual case reports, and a comprehensive Middle Eastern CD40LG variant spectrum has not been established; therefore, population-specific mutational hotspots or genotype–phenotype correlations cannot be inferred from the currently available data.The proband was initially diagnosed with juvenile idiopathic arthritis, while primary immunodeficiency was recognized at two years of age and the molecular diagnosis of X-HIGM1 was established at five years. Inflammatory and autoimmune manifestations, including arthritis, have been reported in CD40L deficiency and may complicate recognition of the underlying immunodeficiency [18]. In this setting, immunosuppressive treatment for inflammatory arthritis should be used with careful monitoring because patients with CD40L deficiency remain susceptible to serious and opportunistic infections [19]. HSCT was recommended by the treating physicians, and outcomes are influenced by age at transplantation and pre-existing organ damage [9]; donor identification is ongoing.

## 5 Limitations

This report has several limitations. CD40L expression by flow cytometry was not assessed because the assay was not available at the time of evaluation, and no direct functional or protein-expression studies were performed; therefore, the functional consequences of the identified variant remain predicted rather than experimentally confirmed. Molecular testing was limited to the available family members, and additional affected or at-risk relatives could not be tested because suitable samples were unavailable. In addition, the clinical histories and causes of death reported for deceased maternal relatives were not molecularly confirmed and therefore should be considered suggestive rather than diagnostic of CD40LG deficiency.

## 6 Conclusion

In summary, we identified a previously unreported hemizygous frameshift variant, CD40LG: c.127del (p.Ala43LeufsTer11), in an Iranian boy with clinical features consistent with X-HIGM. This variant is predicted to result in early protein truncation and loss of normal CD40LG function, consistent with the established loss-of-function mechanism of CD40LG-related disease. Sanger sequencing confirmed the variant in the proband and demonstrated maternal carrier status, with the segregation findings in the tested family members consistent with X-linked inheritance. Prenatal genetic testing was subsequently performed in the family using fetal DNA obtained by amniocentesis. This case expands the known mutational spectrum of CD40LG by adding a previously unreported frameshift variant affecting exon 1. More broadly, it emphasizes the importance of early molecular diagnosis in patients with recurrent infections and suggestive family histories, as accurate genetic identification can inform treatment planning, carrier detection, reproductive counseling, and prenatal testing.

## CRediT authorship contribution statement

**Fatemeh Farzi:** Writing – review & editing, Writing – original draft, Resources, Methodology, Investigation, Formal analysis, Data curation. **Mohammad Panahi:** Writing – review & editing, Writing – original draft, Validation, Software, Methodology, Formal analysis, Conceptualization. **Ebrahim Sakhinia:** Writing – original draft, Visualization, Validation, Software, Resources, Methodology, Conceptualization. **Effat Alizadeh:** Writing – review & editing, Writing – original draft, Resources, Methodology, Investigation, Formal analysis, Data curation, Conceptualization.

## Clinical trial number

Not applicable.

## Consent for publication

Written informed consent for publication of the patient's clinical and genetic information was obtained from the patient's parents or legal guardian.

## Ethics approval and consent to participate

Formal ethics committee approval was not obtained for this single-patient case report. Written informed consent was obtained from the patient's parents or legal guardian for genetic testing and participation in the study.

## Funding statement

No specific funding was received for this study.

## Declaration of competing interest

The authors declare that they have no known competing financial interests or personal relationships that could have appeared to influence the work reported in this paper.

## Data Availability

The clinical and genetic data generated and analyzed during this study are not publicly available because they contain potentially identifiable patient information. Relevant de-identified data supporting the findings of this study are available from the corresponding author upon reasonable request and subject to appropriate ethical and privacy considerations.
